# *In-Situ* Analysis of Essential Fragrant Oils Using a Portable Mass Spectrometer

**DOI:** 10.1155/2019/1780190

**Published:** 2019-04-01

**Authors:** Fred P. M. Jjunju, Stamatios Giannoukos, Alan Marshall, Stephen Taylor

**Affiliations:** ^1^Department of Electrical Engineering and Electronics University of Liverpool, Brownlow Hill, L69 3GJ, UK; ^2^Laboratory of Atmospheric Chemistry, Paul Scherrer Institute, 5232 Villigen, Switzerland; ^3^Q-Technologies Ltd, 100 Childwall Road, Liverpool L15 6UX, UK

## Abstract

A portable mass spectrometer was coupled to a direct inlet membrane (DIM) probe and applied to the direct analysis of active fragrant compounds (3-methylbutyl acetate, 2-methyl-3-furanthiol, methyl butanoate, and ethyl methyl sulfide) in real time. These fragrant active compounds are commonly used in the formulation of flavours and fragrances. Results obtained show that the portable mass spectrometer with a direct membrane inlet can be used to detect traces of the active fragrant compounds in complex mixtures such as essential fragrant oils and this represents a novel in-situ analysis methodology. Limits of detection (LOD) in the sub-ppb range (< 2.5 pg) are demonstrated. Standard samples in the gaseous phase presented very good linearity with RSD % at 5 to 7 for the selected active fragrant compounds (i.e., isoamyl acetate, 2-methyl-3-furanthiol, methyl butanoate, and methyl ethyl sulphide). The rise and fall times of the DIM probe are in the ranges from 15 to 31 seconds and 23 to 41 seconds, respectively, for the standard model compounds analysed. The identities of the fragrance active compounds in essential oil samples (i.e., banana, tangerine, papaya, and blueberry muffin) were first identified by comparison with a standard fragrance compounds mixture using their major fragment peaks, the NIST standard reference library, and gas chromatography mass spectrometry (GC-MS) analysis. No sample preparation is required for analysis using a portable mass spectrometer coupled to a DIM probe, so the cycle time from ambient air sampling to the acquisition of the results is at least 65 seconds.

## 1. Introduction

Fragrances and flavors are a key part of modern food, drinks, cosmetics, aromatherapy, and many other personal hygiene products. The most important activities in the flavor and fragrance industry are quality control, the search for natural sources for novel fragrant compounds, the development of sampling, and analytical technologies [1]. Currently there is no standard analytical method for the online direct analysis and monitoring of the fragrance active components during the processing and quality control stages. Traditional analytical techniques such as solid phase extraction (SPE) [2, 3] and liquid/liquid extraction (LLE) [1, 4, 5] followed by gas or liquid chromatography mass spectrometry (GC-MS or LC-MS) [6] are routinely used in the analysis and quantification of the active components of the fragrance and flavors with exceptional sensitivity and specificity. However, sample collection and transfer to an offsite laboratory is required [7–9]. These steps are labor intensive and time consuming and require skillful sample preparation personnel [7, 10]. There is a clear need for portable analytical technologies that are field ready to allow* in-situ* analysis at the source [11–17]. This is useful not only for quality control, but also for the development of new fragrant products to satisfy the increasing consumer demand [7]. As it will be shown in this study, a portable mass spectrometer combined with a direct inlet membrane (DIM) probe can meet such criteria.

Membrane introduction mass spectrometry (MIMS) has often been utilized in gathering on-line diagnostic data from complex mixtures present in air or water such as those found in reacting chemical streams [18], environmental monitoring [19], and security and pharmaceutical intermediates [20–22]. Exposure of the sample stream to a semipermeable membrane can be used to selectively introduce compounds present in the fluid matrix into the mass spectrometer by pervaporation [18, 23, 24]. The rate of sample transfer across the membrane depends on its solubility, diffusivity and the membrane material [18]. Compounds that are highly soluble on a polydimethylsiloxane (PDMS) membrane, such as the trihalomethanes, can be measured at high parts-per-trillion (ppt) to low parts per billion (ppb) levels [23]. Once the analyte(s) are introduced into the vacuum system, neutral molecules in vapor phase can be ionized by an electron impact ionization source (EI) or glow discharge source (GDEI) [25]. MIMS is thus characterized by its simplicity, speed and sensitivity. However, it has in the past been limited to laboratory settings due to the cost, size, high vacuum, and power requirement required of mass spectrometers [26, 27].

The development of small footprint portable mass spectrometers is analogous to that of portable computing devices. Miniaturized, light weight, low cost mass spectrometers combined with a membrane sampling system could expand applicability. Possible deployments range from space exploration, food safety monitoring [28], forensics [29–33], and environmental monitoring to personal usage [34–38]. Miniaturization of mass spectrometers is a systematic exercise [39], which involves customized and optimized designs of mass analyzers, ion transfer devices, electronics, and vacuum systems [29, 40–42].

Cooks and Ouyang pioneered the on-site analysis of condensed-phase and semivolatile organics in aqueous phase using small footprint miniaturized quadrupole ion trap based systems combined with a membrane inlet probe [40, 43–48]. Other groups have developed portable MIMS systems [49, 50] focusing on the development of portable quadrupole mass filters coupled with membrane inlet sampling for the direct* in-situ* analysis of a wide range of volatile organic compounds (VOCs) and semi-VOCs in both gaseous and aqueous phase [49, 51–55]. Most recently a series of VOCs have been used to encapsulate and modulate data using a portable MS coupled with a direct inlet membrane probe with high sensitivity [56].

*In-situ* analytical methods have several important applications in the food, fragrance industry, quality control and personal hygiene [1, 57]. Here, a small footprint portable mass spectrometer combined with a direct inlet membrane (DIM) probe represents a novel method for on-site and online monitoring of flavors and fragrances [6, 58].

## 2. Experimental Section

### 2.1. Chemicals and Reagents

Organic methanol solvent, (HPLC grade, 99.8%), reference standard model compounds were of analytical grade (3-methylbutyl acetate, 2-methyl-3-furanthiol, methyl butoate, and ethyl methyl sulfide) with similar chemical properties (i.e. aldehydes and esters) as the active compounds used in most flavors and fragrance products, were purchased from Sigma-Aldrich Corporation, (UK) and used without any further purification. All chemicals were neat liquids and were stored at room temperatures prior to their use. A reference artificial standard mixture was prepared using methanol as a solvent at a concentration of 100 *μ*g/mL for each model standard compound. The essential fragrant oils (i.e., tangerine, papaya, banana, and blue berry muffin) samples were bought from Mystic Moments, Hampshire UK (www.mysticmomentsuk.com), and used as supplied without any purification.

### 2.2. Sample Preparation

**Figure 1:**
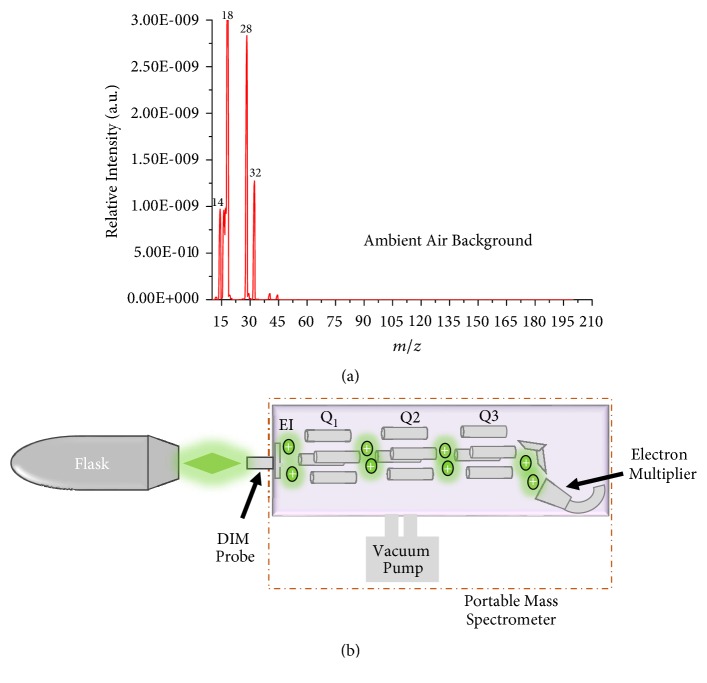
Experimental setup used in the detection of different fragrance formulations using a portable quadrupole mass spectrometer coupled with the a direct membrane (DIM) probe; (a) shows the mass spectra obtained for ambient air background without any sample in the flask and (b) the schematic of the portable mass spectrometer coupled to a DIM probe with a flask placed in front of the probe.

Standard model compounds were dissolved in methanol solvent (HPLC grade) to make a stock solution at 1000 ppm. Working solutions were prepared by serial dilution with methanol. An artificial mixture consisting of each of the model compounds at 100 ppb concentration was prepared so that approximately the same ion abundances might be recorded. From each solution, approx. 5 *μ*L was deposited in a flask (1300 mL) obtained from Sigma-Aldrich UK and then analyzed using a portable mass spectrometer system combined with a DIM probe system as shown in Figure 1. The essential fragrant oil mixtures (i.e., tangerine, papaya, banana, and blue berry muffin) were used as supplied without any modification or pre-concentration. In this case, the headspace vapors emitted from the bottles with the essential fragrant oils (10 mL) were exposed in close proximity to the DIM probe for 2 minutes in the open air.

### 2.3. Portable Mass Spectrometer

Experiments were performed using a portable quadrupole mass spectrometer system (Q-Technologies Ltd., UK), tuned for the optimum continuous detection of the analyte(s) of interest. The main components of the portable mass spectrometer are as follows: a triple filter quadrupole mass spectrometer (consisting of an electron ionization (EI) ion source, mass analyzer, and detector, a vacuum system, an electronics control unit (ECU), and a laptop computer. The mass analyzer is made up of 25 mm rf only prefilter ((Q1) rf only) followed by 125 mm main dc/rf quadrupole mass filter (Q2) and a 25 mm small rf only post filter (Q3) all arranged in series (Figure 1). The portable mass spectrometer has a mass range of* m/z* 1–200 Da with 1 unit mass resolution. The used portable mass spectrometer employs two different types of detectors: (a) a Faraday cup and (b) a Channeltron type electron multiplier. The portable mass spectrometry system is enclosed in a stainless steel chamber pumped down by a TURBOLAB 80 vacuum system obtained (Oerlikon Leybold Vacuum Ltd., Chessington, UK). The TURBOLAB 80 consists of an Oerlikon dual-stage oil-free DIVAC 0.8 T diaphragm pump and a TURBOVAC SL 80 H turbo molecular pump. The overall system pressure was continuously monitored using a digital pressure gauge (model MRT 100) from Pfeiffer Vacuum Ltd. (Newport Pagnell, UK) that uses a Pirani-Cold cathode method of measurement. Total base pressure of the system when the sample inlet valve is fully closed is 2.5 × 10^–8^ Torr. Operating pressure during experiments with the sample valve fully open and the membrane sampling probe attached was stable between 3.0 × 10^–6^ Torr and 4.0 × 10^–6^ Torr. All components of the portable mass spectrometer are housed within a stainless steel vacuum chamber weighing less than 20 kg. Data was acquired using a laptop running Windows 7 operating system and data interpretation was performed using the NIST14 mass spectral library. Multiple ion monitoring mode was used to continuously select characteristic mass fragments for the chemical analyte(s) of interest.

A DIM interface probe was used for preconcentrating and introducing analyte(s) from vapor phase samples in real time through pervaporation [22, 59]. The membrane used was a thin fine non-sterile flat polydimethylsiloxane (PDMS) (Technical Products, Inc. of Georgia, USA) with thickness of 0.12 mm and sampling area of 33.2 mm^2^. The membrane was supported by a 0.8 mm thick stainless steel porous frit with 10 *μ*m porosity. The operation of the DIM probe is shown in Figure 1(b). Exposure of sample to the membrane was achieved by placing an open flask with 5 *μ*L of the sample deposited ~ 2 mm away from the DIM probe. The headspace vapor of the analyte(s) molecules pervaporates through the membrane into the quadrupole mass filter (QMF) under ambient conditions in the open environment. The pervaporated molecules are ionized by electron impact ionization. The distance between the membrane and the (EI) source is 50 mm. Ions are generated from an electron filament biased at 1.6 mA electron emission current and electron energy at 65 eV. The manifold remains closed at all times to maintain a constant pressure. The total response of characteristic ions from the analyte(s) of interest was reconstructed from the total ion current of a full scan mass spectrum in the chromatograph mode. Depending on the analyte(s) being studied the duty cycle of the entire analysis process ranged from 30 to 60 seconds.

The analytical performance of the portable mass spectrometer with a direct inlet membrane (DIM) probe combined was carried out using a static dilution procedure [60, 61]. Liquid stock solutions of 10 mL screw vials (Agilent Technologies LDA UK Ltd) of model standards were prepared in methanol at varying concentrations (250, 500, 1000, and 5000 and 10000 ppb). Approximately 5 *μ*L of each standard were pipetted in 1.3 mL narrow-neck glass flasks (Sigma Aldrich Co. LLC., UK). The glass flasks were carefully lidded with wrapping film (parafilm) to eliminate sample loss. A 3-hour phase of incubation at room temperature (~23°C) was followed to allow complete evaporation of the sample analyte(s) and generation of the gas standards. The glass flasks were carefully cleaned prior to the above process with water, soap, and then deionized water (ReAgent Chemical Services Ltd, Cheshire, UK). The generated gaseous standards were tested using the experiment setup in Figure 1. The mass spectra of the analyte(s) were recorded from the lowest to the highest concentration to avoid possible memory effects.

## 3. Results and Discussion

Results obtained show that the DIM probe combined with a portable experiment retains the advantages of high sensitivity and specificity typical of traditional MS measurements, plus short analysis time (< 1 minute) with no sample pretreatment. Low limits of detection (LOD) of 2.5 pg (absolute concentration) and acceptable reproducibility (RSD of < 10 %) in a variety of untreated, complex essential fragrant oil (i.e., tangerine, papaya, banana, and blue berry muffin) samples were achieved. The strength of the used portable mass spectrometer lies not only in the small size and low weight for in field analysis but also in its ability to be used with a wide range of other mass spectrometry sample inlet methods beyond the DIM probe used in this study [62].

We chose to study different fragrant compounds such as 3-methylbutyl acetate (MW 130), methyl butanoate (MW 102), ethyl methyl sulfide (MW 76), and 2-Methyl-3-furanthiol (MW 114) because they are commonly used as precursors or active ingredients on a large scale in the composition of many flavors and fragrant commercial products due to their intense and pleasant aromatic smell with low toxicity [63, 64]. As such their detection and quantification is vital to ensure high quality production of consumer products in both food and cosmetic industries [65] and aromatherapy treatment [66]. In addition, there is the need to monitor the authenticity and level of additives to combat fragrance product adulteration [67–69]. Before any sample was analyzed, the direct membrane inlet probe with a portable mass spectrometer was first characterized by recording the background mass spectrum of an empty clean flask (i.e., without any sample) as shown in Figure 1. The recorded background mass spectra show highly intense peaks at* m/z* 18, 28, and 32 for water clusters, nitrogen, and oxygen ions, respectively. The analyte(s) were introduced into the vacuum manifold of the portable mass spectrometer through pervaporation using a DIM probe (PDMS membrane) and ionized by EI, producing spectra which show the main features similar to those of the NIST mass spectra reference library recorded using different instruments [70].

**Figure 2:**
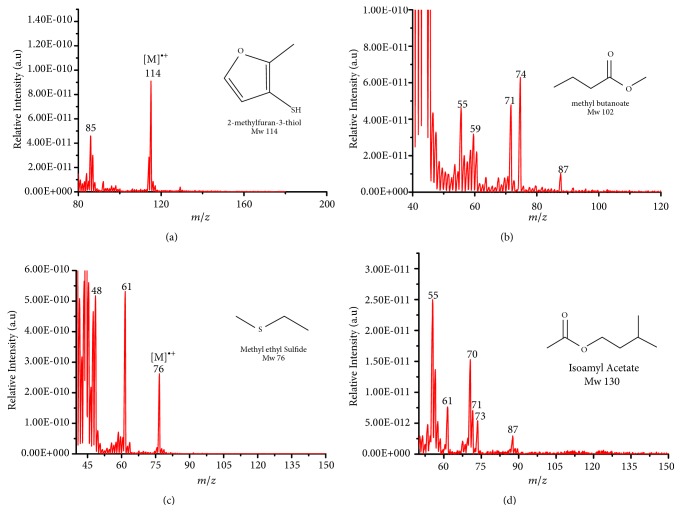
Representative EI mass spectra of fragrant model compounds recorded using a DIM probe combined with a portable mass spectrometer. Accurate amounts of analyte were pitted into a flask and introduced into the manifold of the portable MS through a DIM probe, 5 *μ*L,* viz *10 ppb. (a) 2-methylfuran-3-thiol (Mw 114), (b) methyl butanoate (Mw 102), (c) ethyl methyl sulfide (Mw 76), and (d) 3-methylbutyl acetate (Mw 130).

Representative electron impact (EI) mass spectra for 2-methyl-3-furanthiol are shown in Figure 2. An intense molecular radical cation [M]^+.^ peak at* m/z* 114 of 2-methyl-3-furanthiol with a fragment peak at* m/z* 85 due to the subsequent neutral loss of -[CHO] or –[29 Da] was observed. The identity of 2-methyl-3-furanthiol was confirmed by comparing the NIST mass spectrum for 2-methyl-3-furanthiol with the mass spectrum obtained using the DIM probe coupled to a portable mass spectrometer system [70].  Figure 2(b) shows the recorded mass spectra obtained for methyl butanoate (MW 102). A less intense fragment peak at* m/z* 87 due to the methyl neutral loss -[CH_3_ or -15 Da] followed by a major intense peak at* m/z* 74 due to McLafferty rearrangement [71] – [CO or 28 Da]. This is again followed by *α*-cleavage at* m/z* 71 due to the loss of – [CH_2_OH or 31 Da] further confirming the structure of methyl butanoate (Mw 102). The McLafferty rearrangement observed is as a result of the *β*-cleavage and the transfer of a *γ*-hydrogen [72]. The moderate fragment peak at* m/z* 87 is characteristic of methyl esters which results from *γ*-cleavage. As such the fragment peaks at* m/z* 74 and 87 peaks provide definite confirmation that methyl butanoate detected is a straight chain C_4_-C_26_ methyl ester [72].  Figure 2(c) shows the mass spectrum of ethyl methyl sulfide (MW 76) recorded using a potable mass spectrometer with a direct inlet membrane probe system. An intense molecular cation [M]^+.^ at* m/z* 76 followed by an intense fragment peak at* m/z* 61 due to the neutral loss of methyl radical – [CH_3_ or 15 Da] was observed. The mass spectrum for 3-methylbutyl acetate (MW 130) (Figure 2(d)) shows no molecular ion with a less intense fragment peak at* m/z* 87 due to the apparent neutral loss of -[43 Da] followed by a highly intense fragment peak at* m/z* 76 due to the apparent neutral loss of [-54 Da]. The structure and identity of 3-methylbutyl acetate was confirmed using the mass spectral profile of the standard using the NIST online mass spectra database. The presence of the fragment peak at* m/z* 87 peak provides definite confirmation that 3-methylbutyl acetate is a straight chain ester [73]. Esters with low molecular weight are commonly used as precursors of different flavors and fragrances due to their low toxicity [63], low polarity, and lower boiling point [9, 69].

**Figure 3:**
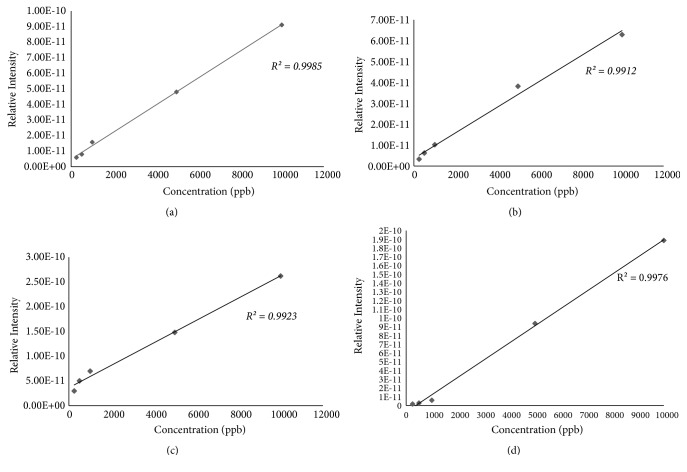
Calibration curve for the standard model compounds using various concentration (250 – 10000 ppb) samples of (a) 2-methyl-3-furanthiol (Mw 114), (b) methyl butanoate (Mw 102), (c) ethyl methyl sulfide (Mw 76), and (d) 3-methylbutyl acetate (Mw 130 obtained from the portable mass spectrometer with a DIM probe.

The analytical performance was evaluated for all the standard fragrant model compounds studied Figure 3.  Figure 3(a) shows the calibration curve for 2-methyl-3-furanthiol, and the system was linear over 4 orders of magnitude (250 to 1000 ppb) with* r*^2^ value of 0.9985, for 60 seconds sample exposure time. The LOD for the standard fragrant model compounds was determined as the concentration that produces a signal more than three times greater than the standard deviation plus the mean value of the blank, in full MS mode. For 2-methyl-3-furanthiol a limit of detection (LOD) of 2.5 pg (Table 1) was obtained when analyzed using DIM probe coupled to a portable mass spectrometer. The signal intensity ratios of the most abundant fragment peaks were found to be linear in the range from 250-10000 ppb (Figure 3).

Limits of detection (LOD) for the standard fragrant model compounds were also established. Table 1 shows the LOD (S/N = 3 in the full scan mass spectrum) and the rise and fall times for fragrant model standards (2-methyl-3-furanthiol, methyl butanoate, ethyl methyl sulfide, and 3-methylbutyl acetate). All the detected model standards gave limits of detection in sub ppb range. The rise and fall time for each compound was also measured, and it was observed that the rise times varied from 15 seconds (3-methylbutyl acetate), 16 seconds (ethyl methyl sulfide), and 23 seconds (methyl butanoate) to 31 seconds (2-methyl-3-furanthiol), and also the fall times varied from 23 s seconds (3-methylbutyl acetate), 38 seconds (methyl butanoate), and 41 seconds (ethyl methyl sulfide) to 46 seconds (2-methyl-3-furanthiol). The duration of the rise and fall times varied depending on the analyte(s) and the difference in the physical properties (vapor pressure at 25°C) at which the analyte(s) pervaporates from the membrane, which is attributed to the difference in volatility and solubility of the analyte(s) through the membrane [27].

### 3.1. In-Situ Analysis of Fragrant Compounds in a Mixture Analysis

**Figure 4:**
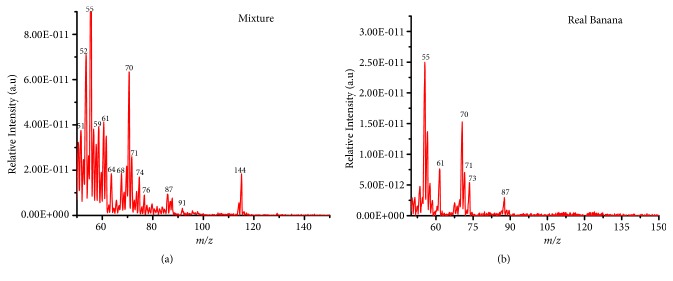
In-situ analysis of standard model compounds in an artificial; (a) mixture of equal volumes of 10 ppm of methylbutyl acetate, 2-mehtyl-3-furanthiol, methyl butanoate, and ethyl methyl sulfide 10 ppm (v/v) using a direct membrane probe coupled to a portable mass spectrometer. Approx. 5 *μ*L of the mixture was deposited in the flask and left for 2 hours. The headspace vapor of the mixture was detected using a portable mass spectrometer coupled to a direct membrane probe and (b) shows the mass spectrum of the real banana headspace vapor measured using DIM probe with a portable mass spectrometer.

Analysis of fragrant components in complex mixtures was also investigated. For these experiments, an artificial mixture was prepared by mixing equal volumes of the model compounds at 10 ppm v/v (i.e., 3-methylbutyl acetate (Mw 130), methyl butanoate (102), ethyl methyl sulfide MW 76), and 2-methyl-3-furanthiol (114)) and analyzed without any further pretreatment. Approximately 5 *μ*L of the artificial mixture was deposited into the vacuum flask and analyzed directly. Figures 4(a) and 4(b) shows the recorded mass spectrum for the artificial mixture and the real banana, respectively. An intense radical molecular cation [M]^+.^ peak at* m/z *114 of 2-methyl-3-furanthiol and a moderate radical molecular cation [M]^+.^ peak at* m/z *76 for ethyl methyl sulfide were observed, while for methylbutyl acetate (Mw 130), two intense fragments at* m/z* 55 and 76 and methyl butanoate (102) and 55, 71, and 74 fragments observed were similar to those in Figures 2(b) and 2(c).

**Figure 5:**
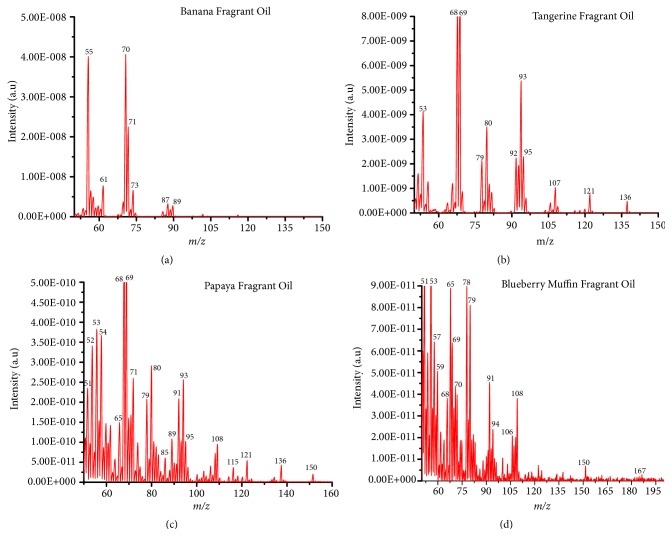
Electron impact ionization mass spectrum for essential fragrant oils analyzed using a direct membrane probe coupled to portable mass spectrometer. (a) banana, (b) tangerine, (c) papaya, and (d) blueberry muffin. Approx. 5 *μ*L of each was deposited in the flask and left for 2 hours forming a headspace vapor and analyzed for 2 minutes with 70 eV EI energy.

The ability to detect the individual standard compounds in artificial mixtures encouraged us to apply this experiment to the analysis of the commercial fragrant mixtures using a portable mass spectrometer coupled with DIM probe. For this experiment, 4 essential fragrant oils (i.e., tangerine, papaya, banana, and blue berry muffin) in their pure form were analyzed with DIM probe combined with a portable mass spectrometer.  Figure 5 shows the recorded mass spectra for the fragrant essential oils samples analyzed. A summary of the detected compounds in the essential oil samples (i.e., banana, tangerine, papaya, and blue berry muffin) is shown in Table 2. Again the mass spectra recorded showed typical characteristic fragment peaks at* m/z* 43, 76, and 87 indicating the presence of esters in the mixture that corresponds to the loss of -[CHO] or –[29 Da] methyl neutral loss – [CH_3_ or 15 Da] as observed with the single model compounds (Figure 2).

To confirm the molecular structure of the compounds identified, GC-MS analysis for the essential fragrant oil in question was undertaken (for experimental procedure see supporting information). Representative GC-MS total ion chromatograms for the different compounds analyzed (tangerine, papaya, banana, and blueberry muffin) are shown in Figures S1–S4 (supporting information). The chromatogram reports the ion current corresponding to the mass spectra of compounds that were later confirmed using the NIST reffernce library matching (Figures S1–S4). Table 2 summarizes the list of compounds identified using the DIM probe with a portable mass spectrometer and confirmed using GC-MS experiments (supporting information). The GC-MS data acquired for complex essential fragrant oil samples provides definite confirmation for the different compounds in the essential fragrant oil samples identified using a DIM coupled to portable mass spectrometer experiment. It is important to note that the portable mass spectrometer has a lower mass range (1-200 Da) than the GC-MS limiting its capacity to analyze high molecular weight compounds (see supporting information for more details). For instance, diethyl phthalate (Mw 222) a well-known lower molecular weight endocrine disruptor was found in all the essential fragrant samples studied but was not detected using the DIM probe with a portable mass spectrometer owing to its lower mass range. However, this was detected in all the tangerine, papaya, and blueberry muffin essential oil samples when analyzed with GC-MS (see supporting information for more details).

The results obtained show the capability of the portable mass spectrometer for online high-throughput rapid screening of different analyses with no sample preparation. However, the use of electron impact (EI) ionisation complicated the mass spectra of the analyte(s) studied. EI ionisation can generate ions with high internal energies (70 eV), hence in-source fragmentation is common and can complicate the interpretation and identification of the fragrant components in the mixture. To overcome this problem softer ionisation techniques (e.g., chemical ionisation (CI)) or atmospheric pressure chemical ionisation (APCI) or other ambient ionisation sources forming ions with low internal energies suppressing fragmentation can be used in unison with the portable mass spectrometer [34, 39, 74].

In this study the direct analysis of different fragrant active in complex mixtures has been demonstrated using a DIM probe coupled with a portable mass spectrometer. The results demonstrate rapid analysis allowing high throughput of different essential oil fragrant compounds of importance in the flavor and fragrance industry. The results reported that a DIM probe coupled to a portable mass spectrometer can be incorporated during in the production and the post production stages of in different flavors in the fragrance industry for quality control.

Quality control in the flavour and fragrance industry has become a global problem. Being a useful analytical tool for in-situ analysis at the source with minimal sample preparation, a DIM probe coupled with a portable mass spectrometer is proposed as the alternative analytical technique to the analysis of flavours and fragrant samples in complex matrixes such as fragrant essential oils. As demonstrated, DIM probe combined with small footprint mass spectrometer is able to detect the active components of different food flavours and fragrant essential oil with high throughput. Linear signal responses with a dynamic range of 5 orders of magnitude were obtained. The limits of detection (LOD) were 2.5 pg (absolute concentration) with good reproducibility (RSD < 10 %). The rise times of 16 to 31 seconds and fall times of 23 to 41 seconds are noteworthy providing a timely and direct analysis of different flavours and fragrants. Future work will involve optimising the membrane parameters to enhanced performance. Because no sample preparation is needed for the analysis, the duty cycle time from ambient air sampling to acquisition of results is 65 seconds or less.

The simplicity and the ability to analyze different flavors and fragrant samples using a direct membrane probe further enhance the potential of using a portable or miniaturized mass spectrometer for online monitoring, during the production and formulation of the flavors and fragrances, and for forensics investigation. Such a system in operation would be of great value in the flavor and fragrant industry for online monitoring and quality control. The data obtained can also be used in aromatherapy or olfactory experiments in a range of environments. Future work will involve the coupling of softer ionisation source operating at atmospheric pressure such as desorption atmospheric pressure chemical ionisation (DAPCI) for a wide range of food flavours.

## Figures and Tables

**Table 1:** Limit of detection and rise and fall times for the fragrant compounds analyzed.

Compound	Vapor Pressure	Exposure	Ions	^x^LOD	Rise	Fall
	(mm Hg at 25°C)	Time (s)	Monitored (*m/z*)	(ppb)	Time (s)	Time (s)
2-methyl-3-furanthiol	5.78	60	114	8.38	31	46
Methyl butanoate	32	65	74	15.06	23	38
ethyl methyl sulfide	60	60	76	1.68	16	41
3-methylbutyl acetate	5.6	50	70	16.23	15	23

^xx^Limit of detection (LOD) was calculated as = 3.3(standard error/slope), taken from a calibration curve of five points in the range 250-1000 ppb with three repetitions for each point.

**Table 2:** Compounds detected in the essential oil fragrant samples using a DIM probe coupled to a portable mass spectrometer.

Essential Fragrant Oil	Identified compounds	Chemical Structure	Molecular Weight	Ions Detected	Major Product Ions (*m/z*)
Tangerine	*β*-Myrcene	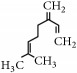	136	[M]^.+^	93,79, 69,41
	D-Limonene	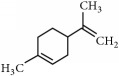	136	[M]^.+^	121.107,93,79,68
	*γ*-Terpinene	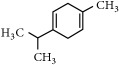	136	[M]^.+^	121, 105, 93, 77
	Terpinolene	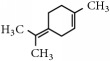	136	[M]^.+^	121,105,93,91,79,77

Papaya	D-Limonene	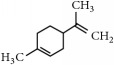	136	[M]^.+^	121, 107, 93, 79 68
	Acetic acid, phenylmethyl ester	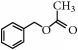	150	[M]^.+^	108, 91, 79
	4-tert-Butylcyclohexyl acetate	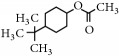	198	n/a	138,123,82,67,57,43
	2(3H)-Furanone, 5-heptyldihydro	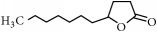	184	n/a	85
	Octanal, 2-(phenylmethylene)	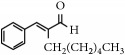	216	n/a	145,129, 115, 91

Banana	Isoamyl acetate	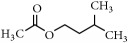	130	n/a	87, 70, 55, 43
	Propanoic acid, 2-methyl-, 3-methylbutyl ester	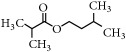	158	n/a	89, 71, 55, 43
	Butanoic acid, 2-methylbutyl ester	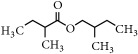	172	n/a	71, 55, 43

Blueberry Muffin	Acetic acid, phenylmethyl ester	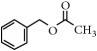	150	n/a	108, 91, 79, 65, 51
	4-tert-Butylcyclohexyl acetate	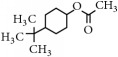	198	n/a	138,123,82,67,57,43
	Isopropyl myristate	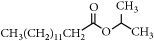	270	n/a	129, 102,60,57,43

[M]^.+^= Molecular radical cation, n/a = Not available (not detected) using DIM probe with a portable mass spectrometer.

## Data Availability

The data used to support the findings of this study are available from the corresponding author upon request.

## Supplementary Materials

Supplementary Materials The mass spectrum figure(s) data used to support the findings of this study are included within the article. Additional information/data used to support the findings of this study are included within the supplementary information: GC-MS experimental procedure, analysis of Banana fragrant Essentials oil using GC-MS, analysis of Tangerine fragrant essential oil using GC-MS, analysis of papaya fragrant essentials using GC-MS, and analysis of blueberry muffin fragrant essentials using GC-MS. The Supporting Information is available free of charge.
