# Supplementary material for: In-Situ Analysis of Essential Fragrant Oils Using a Portable Mass Spectrometer
Source: Int J Anal Chem. 2019 Apr 1;2019:1780190. doi: 10.1155/2019/1780190 (PMC6463677; doi:10.1155/2019/1780190)
Supplement: Supplementary Materials — The mass spectrum figure(s) data used to support the findings of this study are included within the article. Additional information/data used to support the findings of this study are included within the supplementary information: GC-MS experimental procedure, analysis of Banana fragrant Essentials oil using GC-MS, analysis of Tangerine fragrant essential oil using GC-MS, analysis of papaya fragrant essentials using GC-MS, and analysis of blueberry muffin fragrant essentials using GC-MS. The Supporting Information is available free of charge. [file 1780190.f1.pdf]

Supporting Information

In-situ analysis of Essential fragrant oil using a Portable Mass Spectrometer

Fred. P. M. Jjunju[¥*], Stamatios. Giannoukos[¥+], Alan. Marshall[¥], Stephen. Taylor [€]

[¥]Department of Electrical Engineering and Electronics University of Liverpool, Brownlow Hill, L69 3GJ, UK

[€]Q-Technologies Ltd, 100 Childwall Road, Liverpool L15 6UX, U.K

[+]Laboratory of Atmospheric Chemistry, Paul Scherrer Institute, 5232 Villigen, Switzerland

*To whom correspondence should be addressed:

Dr. Fred P. M Jjunju, Department of Electrical Engineering and Electronics Liverpool University, Brownlow Hill L69 3GJ UK, Telephone: 0151 794 4517. Fax: +44(0)1517944540. Email: fjjunju@liv.ac.uk.

Summary of the Supporting Information

Topic	Title of Topic	Page
**Topic 1**	GC-MS Experimental procedure	**3**
**Topic 2 (Figure S1)**	Analysis of Banana fragrant Essentials oil using GC-MS	**4**
**Topic 2 (Figure S2)**	Analysis of Tangerine fragrant Essential oil using GC-MS	**5**
**Topic 2 (Figure S3)**	Analysis of Papaya fragrant Essentials using GC-MS	**6**
**Topic 2 (Figure S4)**	Analysis of Blueberry muffin fragrant Essentials using GC-MS	**7**

Gas Chromatography Mass Spectrometry experimental procedure.

Essential fragrant oils were analyzed using a 5977A quadrupole mass spectrometer (Agilent Technologies, Manchester, UK) equipped with a 7890B gas chromatograph (GC) system (Agilent Technologies, Manchester, UK). The gas chromatographic conditions used were the following: column HP-5MS-UI (30m x 0.250mm x 0.25um); He flow: 1 mL/min; Oven ramp: T1 = 60ºC for 5 min to T2 = 340ºC with temperature step rise 3ºC/min. Essential fragrant oil components were first identified by comparison with a standard fragrance compounds mixture. Representative chromatograms obtained from the GC-MS analysis on standard mixtures are reported in Figure S1-S6. The areas related to

chromatographic peaks due to characteristic ions of the detected components of the essential fragrant oil samples (i.e. banana, targrine, papaya and blueberry muffin), obtained through the reconstructed ion current (RIC) were considered. Representative ion chromatograms obtained from the GC-MS analysis of real essential fragrant oils are shown in Figure S1- S4.

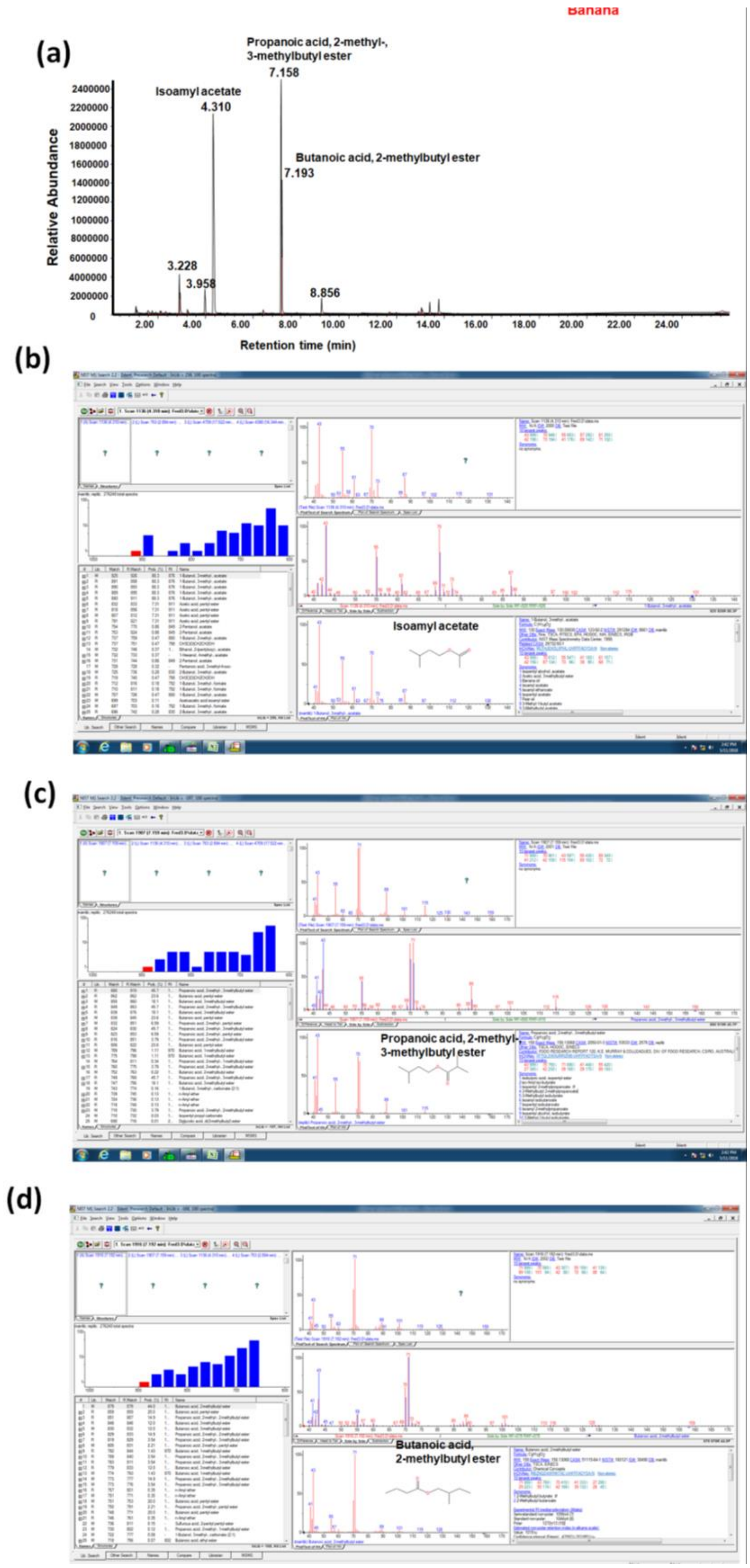

Figure S1. GC-MS analysis of fragrant essential oil; (a) representative chromatogram of banana fragrant essential oil. The chromatogram shows the ion peaks corresponding to the mass spectra of ; (b) isoamyl acetate, (c) propanoic acid, 2-methyl-,3methylbutyl ester, and (d) butanoic acid.

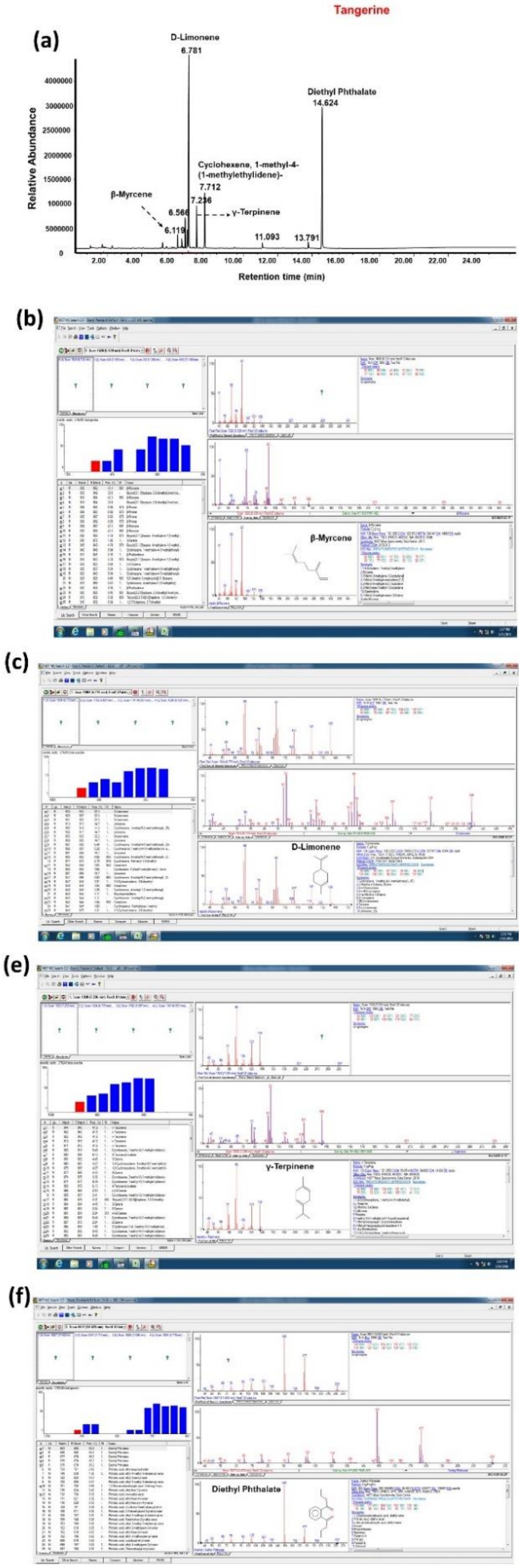

Figure S2. GC-MS analysis of fragrant essential oil; (a) representative chromatogram of tangerine fragrant essential oil. The chromatogram shows the ion peaks corresponding to the mass spectra of; (b) *β*-myrcene, (c) D-limonene, and (d) γ-terpine, (e) terpinolene and (f) diethyl phthalate.

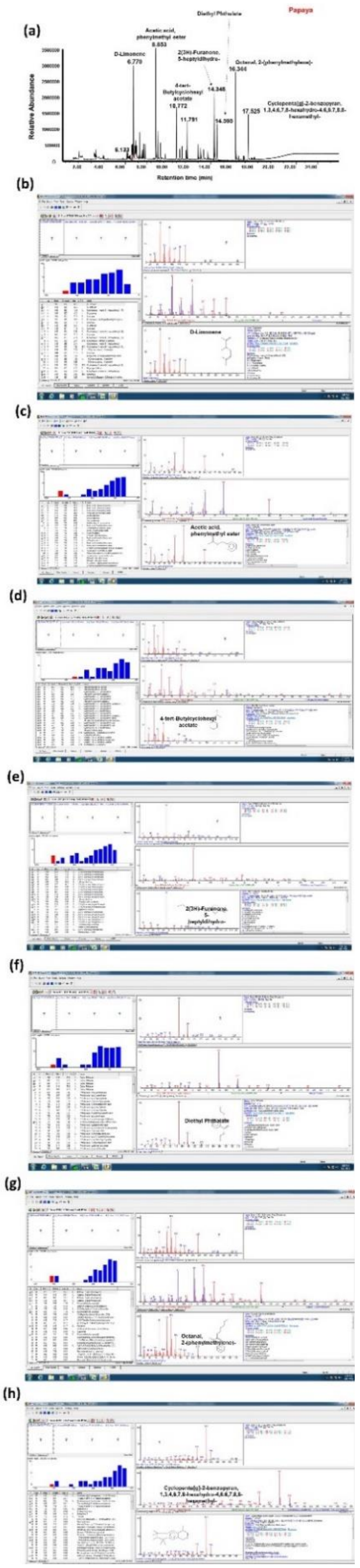

Figure S3. GC-MS analysis of fragrant essential oil; (a) representative chromatogram of papaya fragrant essential oil. The chromatogram shows the ion peaks corresponding to the mass spectra of; (b) D-limonene, (c) acetic acid phenylmethyl ester, (d) 4-*tert*-butylcylohexyl acetate, (e) 2(3H)-Furanone,5-

heptyldihydro , and (f) octanal, 2-(phenylmethylene), (g) Cyclopenta[g]-2-benzopyran, 1,3,4,6,7,8-hexahydro-4,6,6,7,8,8- hexamethyl- and (h) Octanal,2-(phenylmethylene).

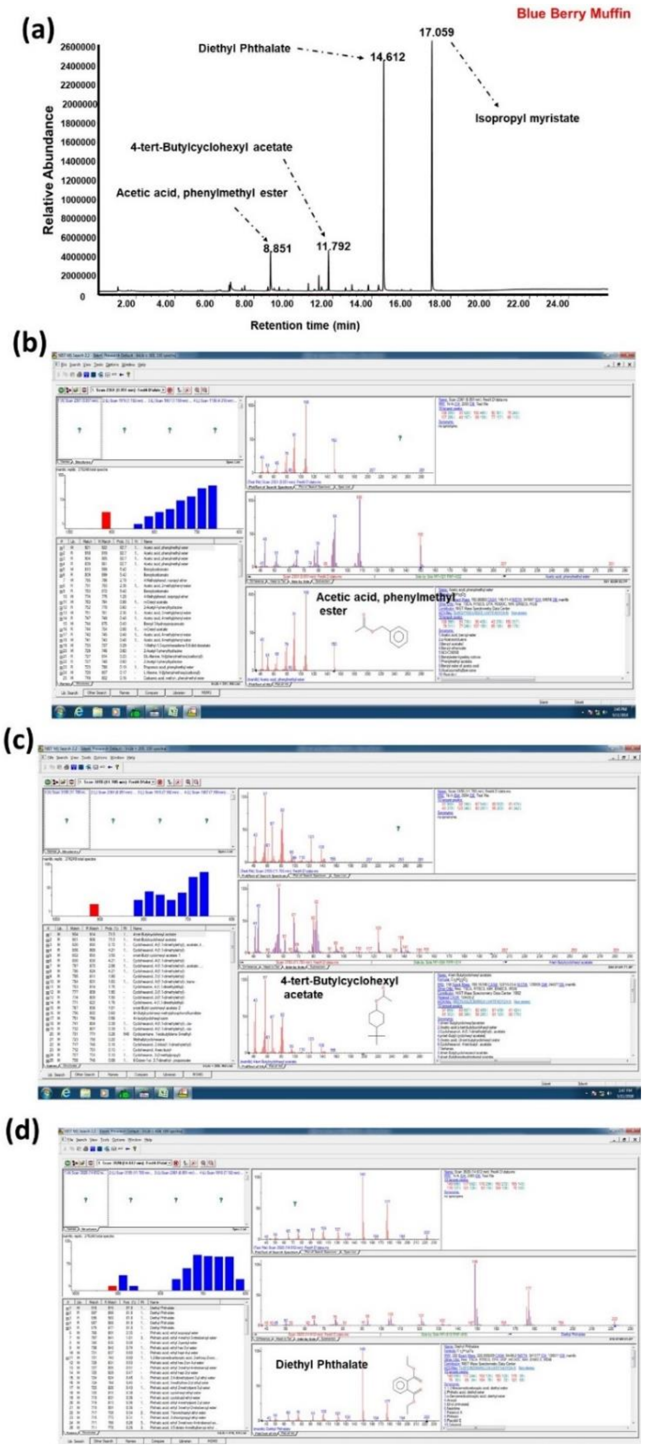

Figure S4. GC-MS analysis of fragrant essential oil; (a) representative chromatogram of blueberry muffin fragrant essential oil. The chromatogram shows the ion peaks corresponding to the mass spectra of; (b) acetic acid, 2-phenylmethyl ester, (c) 4-*tert*-butylcylohexyl acetate and (d) diethyl phthalate.
